# Synthesis and Characterization of SPIONs Encapsulating Polydopamine Nanoparticles and Their Test for Aqueous Cu^2+^ Ion Removal

**DOI:** 10.3390/ma16041697

**Published:** 2023-02-17

**Authors:** Giulia Siciliano, Antonio Turco, Anna Grazia Monteduro, Elisabetta Fanizza, Alessandra Quarta, Roberto Comparelli, Elisabetta Primiceri, M. Lucia Curri, Nicoletta Depalo, Giuseppe Maruccio

**Affiliations:** 1 Department of Mathematics and Physics “Ennio De Giorgi”, University of Salento, Via per Monteroni, 73100 Lecce, Italy; 2 Institute of Nanotechnology, CNR-Nanotec, Via per Monteroni, 73100 Lecce, Italy; 3 Omnics Research Group, Via per Monteroni, 73100 Lecce, Italy; 4 Department of Chemistry, University of Bari, Via Orabona 4, 70126 Bari, Italy; 5 Institute for Chemical and Physical Processes, CNR-IPCF SS Bari, Via Orabona 4, 70126 Bari, Italy

**Keywords:** magnetic iron oxide nanoparticles, polydopamine, copper, remediation

## Abstract

The removal of pollutants, such as heavy metals, aromatic compounds, dyes, pesticides and pharmaceuticals, from water is still an open challenge. Many methods have been developed and exploited for the purification of water from contaminants, including photocatalytic degradation, biological treatment, adsorption and chemical precipitation. Absorption-based techniques are still considered among the most efficient and commonly used approaches thanks to their operational simplicity. In recent years, polydopamine-coated magnetic nanoparticles have emerged for the uptake of heavy metals in water treatment, since they combine specific affinity towards pollutants and magnetic separation capacity. In this context, this work focuses on the synthesis of polydopamine (PDA)-coated Super Paramagnetic Iron Oxide Nanoparticles (PDA@SPIONs) as adsorbents for Cu^2+^ ions, designed to serve as functional nanostructures for the removal of Cu^2+^ from water by applying a magnetic field. The synthetic parameters, including the amount of SPIONs and PDA, were thoroughly investigated to define their effects on the nanostructure features and properties. Subsequently, the ability of the magnetic nanostructures to bind metal ions was assessed on Cu^2+^-containing solutions. A systematic investigation of the prepared functional nanostructures was carried out by means of complementary spectroscopic, morphological and magnetic techniques. Inductively coupled plasma atomic emission spectroscopy (ICP-AES) measurements were performed in order to estimate the Cu^2+^ binding ability. The overall results indicate that these nanostructures hold great promise for future bioremediation applications.

## 1. Introduction

Nowadays, research on contaminants in groundwater and drinking water, including heavy metals, aromatic compounds, dyes, pesticides and pharmaceuticals, is receiving significantly increasing attention due to their effects on human health. Many pollutants are not biodegradable and extremely toxic and can accumulate in living organisms, meaning, ultimately, that they are dangerous, not only for the environment but also for human health, as they may be responsible for several diseases, including cancer, neurological disorders, respiratory diseases and anemia [1,2,3,4]. Many physical-chemical methods have been developed and applied for removing water pollutants, including adsorption, photocatalytic degradation, ion exchange, biological treatment, membrane separation, solvent extraction and chemical precipitation [3,4,5,6,7,8,9]. Among the different approaches, adsorption is one of the most used methods due to its cost effectiveness, easy operation and high efficiency for the treatment of water contaminated by heavy metals and other emergent pollutants, including phenolic compounds, drugs and dyes [10,11,12,13]. In particular, several adsorbents have been developed and applied for the removal of heavy metals, such as carbon nanotubes [14], synthetic porous materials [15] and functionalized polymers [16]; however, all of them are characterized by limited adsorption capacity and lack efficiency in fast separation.

In recent years, magnetic iron oxide nanoparticles (NPs) have received great attention as adsorbents in the field of environmental remediation, thanks to their high surface-area-to-volume ratio that makes them extremely efficient in removing a large amount of pollutants from water in a short time. Furthermore, their superparamagnetic properties allow one to collect these pollutants by applying an external magnetic field, and once the applied magnetic field is removed, the NPs can be easily re-dispersed as they retain no residual magnetism [17,18]. Although many hybrid magnetic nanomaterials have been developed for the removal of water pollutants, the preparation of effective magnetic materials by simple preparation methods is still a challenge, since they need to be properly surface functionalized with different groups to improve their selectivity towards specific analytes [19].

Most recently, polydopamine-coated magnetic NPs [20] have been used for chemical analysis [21] and bio separation [22,23]. Dopamine, a mimic of 3, 4-dihydroxy-L-phenylalanine (L-DOPA), the adhesive foot protein secreted from mussels has, thus, been increasingly studied for the surface functionalization of many inorganic and organic materials, since it can spontaneously polymerize under basic conditions to form polydopamine (PDA), a compund with great adhesive capacity and featuring a relevant functional group suited for further interaction with the species to be absorbed [24]. Recently, such an ability has been extensively applied in forming a hydrophilic firmly adhering coating on NPs with different shapes and surface characteristics, thus accomplishing a versatile and general surface modification strategy due to its mild polymerization property, its hydrophilic characteristics and the abundance of surface functional groups, such as amino, carbonyl and hydroxyl groups, that are able to specifically interact with the relevant substrates [25,26,27].

**Figure 1:**
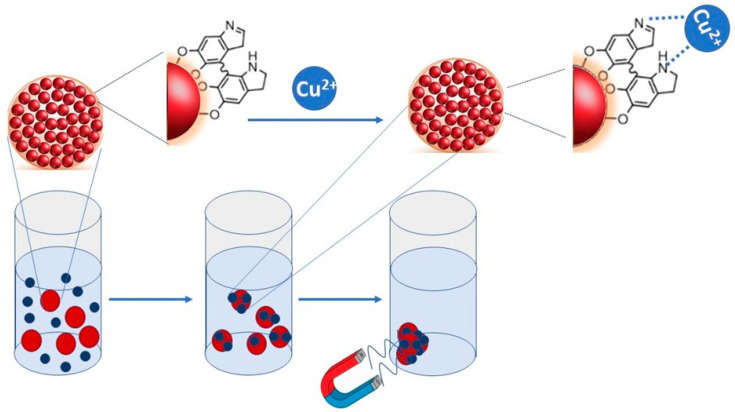
Sketch depicting the adsorption mechanism and the polyvalent coordination of copper due to PDA interaction.

Polydopamine-derived structures, indeed, may incorporate many functional groups, such as amine, catechol and imine, and, thus, establish various interactions, such as hydrogen bonds, chelation, π-π stack and Van der Waals forces, with almost all types of substrates [28]. This singular feature has been exploited in environmental remediation to interact with and remove organic pollutants and metal ions, such as Mn^2+^, Cu^2+^ and Zn^2+^ from water [28,29]. The catechol moieties in PDA possess redox activity that can be used for transition metal binding, leading to the fabrication of diverse hybrid materials with a significant reducing capability towards metal ions [30]. Moreover, PDA can also be used in extreme environments, due to its high chemical stability. Therefore, the combination of chemical properties of PDA with magnetic properties of magnetic NPs resulted in a great interest for adsorption applications, especially as an adsorbent for pollutants [31,32,33,34]. Several works report the preparation and use of Fe_3_O_4_@PDA micro spheres and their application for the removal of heavy metals [31,32,33,34]. Here, we report the synthesis and characterization of PDA-coated Super Paramagnetic Iron Oxide Nanoparticle clusters (SPIONs@PDA NPs), as a function of experimental preparative parameters, and applied as an adsorbent for Cu^2+^ ions, to exploit them as possible functional nanostructures for the uptake of Cu^2+^ from water via the application of an external magnetic field (Figure 1). The work took into account copper as a model contaminant, as its contamination represents a critical environmental issue owing to its extensive use in industrial and agricultural fields, thus urging the development of environmentally friendly solutions characterized by high adsorption capacity and selectivity for an effective sequestration of the Cu from industrial and agricultural wastewater [35].

Here, SPIONs@PDA core–shell NPs were obtained via self-polymerization of dopamine on SPIONs previously synthetized by a thermal decomposition route by using coordinating agents The characterization of the starting SPIONs and of the prepared SPIONs@PDA NPs was carried out by means of complementary spectroscopic, morphological and magnetic techniques, pointing out the role played by the preparative conditions, such as PDA concentration and SPION content, on the final characteristics of the NPs that were finally tested for the copper ion removal from aqueous matrices. For evaluation, effective Cu^2+^ ion binding ability of PDA inductively coupled plasma atomic emission spectrometry (ICP-AES) measurements were performed. The obtained SPIONs@PDA NPs were characterized by a regular and homogenous morphology, superparamagnetic behavior and a good adsorption capacity for Cu(II), as calculated from the fitting of the Langmuir isotherm model. Further optimization of the absorbent properties of the system needs to be addressed to improve its removal efficiency in order to meet the technological requirements for their real application.

## 2. Materials and Methods

### 2.1. Materials and Chemicals

Iron(0)pentacarbonyl (Fe(CO)_5_, liquid 99.99%), 1-octadecene (ODE, technical grade 90%), oleic acid (OLEA, technical grade 90%), oleylamine (technical grade 70%), 1,2-dodecanediol (DDIOL, technical grade 90%), dopamine hydrochloride ((HO)_2_C_6_H_3_CH_2_CH_2_NH_2_HCl, powder), copper(II) sulfate pentahydrate (CuSO_4_•5H_2_O, powder), low metal trace 70% nitric acid and phosphotungstic acid solution (10% *w*/*v*) were purchased from Sigma-Aldrich and used as received. All solvents, namely chloroform, hexane, isopropanol and acetone, were also purchased from Sigma-Aldrich and were of the highest purity available. Phosphate-buffered saline (PBS), tris(hydroxymethyl) aminomethane hydrochloride (Tris-HCl) and borate buffers were prepared by using reagent-grade salts purchased from Sigma. All aqueous solutions were prepared by using water obtained from a Milli-Q Gradient A-10 system (18.2 MΩ cm, organic carbon content ≤ 4 µg L^−1^, Merck Millipore, Darmstadt, Germany).

### 2.2. Synthesis of PDA-Functionalized SPIONs

Organic-capped superparamagnetic nanoparticles (SPIONs) were synthesized by exploiting the thermal decomposition of iron pentacarbonyl precursor in the presence of stabilizer agents according to an experimental procedure previously reported in the literature [32]. The as-synthesized SPIONs, recovered as a black powder, were dispersed in chloroform at final concentration of 12 mg/mL.

Core–shell SPION@PDA NPs were prepared by mixing defined amounts of the as-synthesized SPIONs dispersed in water and dopamine hydrochloride in TRIS-HCl buffer (10 mM, pH 8.5) at a final volume of 1.2 mL, without using any additive. The resulting mixture was kept overnight under vigorous stirring at room temperature. Finally, the SPION@PDA NPs were centrifugated at 3000 RPM for 10 min, rinsed with water and dispersed into MilliQ water.

### 2.3. Copper Ion Adsorption Experiments

For the adsorption studies, a Cu^2+^ solution (10 mL at concentration of 4 ppm and pH = 6) was prepared. Then, different amounts of the SPION@PDA NPs were introduced in the Cu^2+^-containing solution and stirred at room temperature. At specific time points (0, 7, 15, 30, 60, 180, 240 and 480 min, respectively), 500 µL of the as-prepared dispersion was collected and digested at room temperature overnight in low metal trace 70% nitric acid for elemental analysis using a Varian 720-ES ICP-AES. The calibration curve of Cu was prepared in a 0.5–10 ppm range.

For each sample, the time-dependent removal efficiency and the equilibrium adsorption capacity were calculated by Equations (1) and (2), respectively:

(1) $$removal\;efficiency\;\left(\%\right)=\left[(C_{0}-C_{t})/C_{0}\right]\times 100$$

(2) $$adsorption\;capacity,\;\;q_{e}\;\left(mg/g\right)=\left[(C_{0}-C_{e})/W\right]\times V$$

where *C*_0_ and *C_t_* are the initial concentration of Cu^2+^ ions and the concentration at a given time *t* (minutes), expressed in ppm (mg/Kg), respectively, *q_e_* is the adsorption capacity (mg/g) at the equilibrium, *C_e_* is the Cu^2+^ concentration expressed in ppm at equilibrium conditions, *V* is the volume of the solution in liters and *W* is the weight of SPION@PDA NPs expressed in grams.

### 2.4. Transmission Electron Microscopy Analysis

Transmission Electron Microscopy (TEM, Jeol-Jem-1011 microscope), working at an accelerating voltage of 100 kV and equipped with a Quemesa Olympus CCD 11 Mp Camera, was utilized for the morphological characterization of as-synthesized SPIONs and SPION@PDA NPs. Organic-capped SPIONs, dispersed in chloroform, were deposited on 300-mesh carbon-coated Cu grid by dipping, while 5 µL of SPION@PDA NPs dispersed in ultrapure water was deposited by casting. Statistical analysis using freeware Image J analysis program was performed to evaluate average size of the “as synthesized” SPIONs. Staining of SPION@PDA NP samples was achieved by using an aqueous phosphotungstic acid solution 2% (*w*/*v*).

### 2.5. Dynamic Light Scattering and ζ-Potential Analysis

A Zetasizer-Nano S from Malvern operating with a 4 mW He-Ne Laser (633 nm wavelength) was used for the characterization of the SPION@PDA NP samples by Dynamic Light Scattering (DLS) and ζ-Potential measurements. Size distribution is described in terms of polydispersity index (PDI). All samples were diluted by using ultrapure water and filtered (Cameo™ Syringe Filter cellulose acetate membrane, pore size 0.45 μm, diam. 17 mm, Sigma-Aldrich, Darmstadt, Germany) to remove any interfering dust particles. Three measurements were acquired for each sample.

### 2.6. Magnetic Characterization

The magnetic properties of the SPION@PDA NP samples were investigated by using a vibrating sample magnetometer (VSM, Cryogenic Ltd, London, UK). The magnetization curves (M-H) were acquired at 300 K over the magnetic field range (−10 kOe and 10 kOe). The measurements were performed on dry samples and the recorded magnetization curves were normalized to the amount of iron detected via the ICP-AES technique. To this end, a calibration curve of Fe in the 0.5–5 ppm range was set.

### 2.7. ATR-FTIR Absorption Characterization

Mid-infrared spectra were acquired with a PerkinElmer Spectrum One Fourier-transform infrared (FTIR) spectrometer equipped with a DTGS (deuterated triglycine sulfate) detector. For attenuated total reflection (ATR) measurements, the internal reflection element (IRE) was a three-bounce, 4 mm-diameter diamond microprism. NP films were cast directly onto the internal reflection element by depositing the solution of interest (3–5 µL) on the upper face of the diamond crystal and allowing the solvent to evaporate completely. The spectroscopic measurements on NP solutions were carried out at room temperature.

## 3. Results

### 3.1. PDA-Functionalized SPIONs: Preparation and Characterization

**Figure 2:**
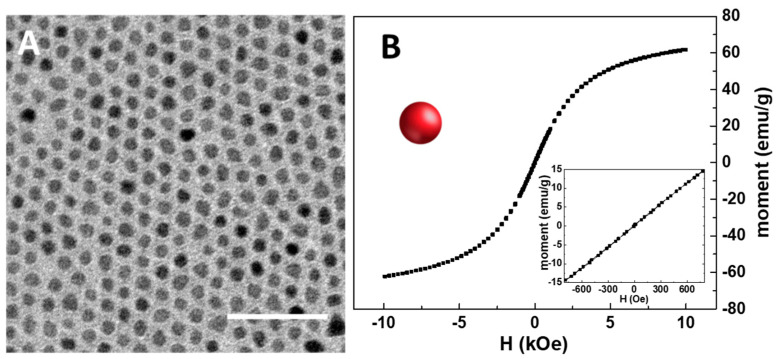
TEM micrograph of the organic-capped SPIONs dissolved in chloroform (scale bar 50 nm) (**A**). RT hysteresis cycle of organic-capped SPIONs (**B**). In the inset, details of the low field magnetization response are reported.

As a preliminary step in the preparation of the SPION@PDA NPs, the synthesis of organic-capped SPIONs was carried out at 250 °C to promote the hot decomposition of iron pentacarbonyl an as iron precursor in the presence of coordinating solvents, such as OLEA and OLEAM, which coordinate the surface of the NPs, also directing their growth [36]. This synthetic procedure best allows one to have a good control of the size, crystallinity and, accordingly, the resulting physicochemical properties of SPIONs. After purification, the as-synthesized SPIONs, having OLEA and OLEAM molecules as capping agents, were characterized in terms of morphological and magnetic properties. The TEM micrograph reported in Figure 2A reveals the formation of SPIONs characterized by mostly spherical shape and size homogeneity, with an average diameter of (7.5 ± 0.5) nm, thus indicating the formation of NPs with very narrow size distribution. Interestingly, a very uniform ordered distribution of SPIONs on the TEM grid, characterized by a regular interparticle distance, can be observed, due to the presence at the SPION surface of the OLEA and OLEAM molecules that, interdigitating, leave the NPs well isolated and arranged [37] (Figure 2A). A magnetic moment of about 60 emu g^−1^ at 10 kOe was recorded, in agreement with the literature findings [38,39]. In addition, the symmetrical sigmoidal shape of the magnetization curves and the absence of remanence and coercitivity (see the inset in Figure 2B) confirm the superparamagnetic behaviour of the synthesized SPIONs, as expected for their small dimension.

**Figure 3:**
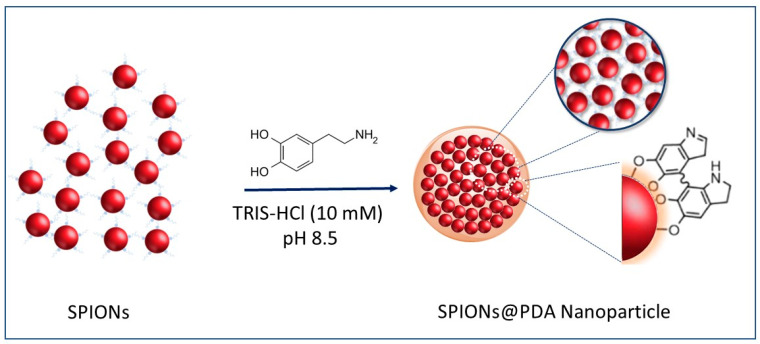
Representative sketch of the preparation of the SPION@PDA NPs.

The preparation of SPION@PDA NPs was performed according to a procedure previously reported by H. Hu et al. [40] (see the Scheme of Figure 3). In detail, the formation of SPION@PDA NPs was achieved by exploiting an ex situ procedure that promotes the adsorption of PDA on the surface of SPIONs. The formation of electrostatic and hydrophobic interactions as well as hydrogen bonds allows for a fine control of the nanocomposite morphology [41]. In particular, a PDA layer was formed onto the SPION surface through self-polymerization of dopamine in the Tris-HCl buffer solution [28]. Indeed, the catechol groups, responsible for the adhesion to substrates, are oxidized to o-quinone, ensuring the formation of a cross-linked shell [42]. In addition, the hydroxyl groups on the SPION surface may also interact with the catechol functionality of dopamine through dehydration [43].

**Figure 4:**
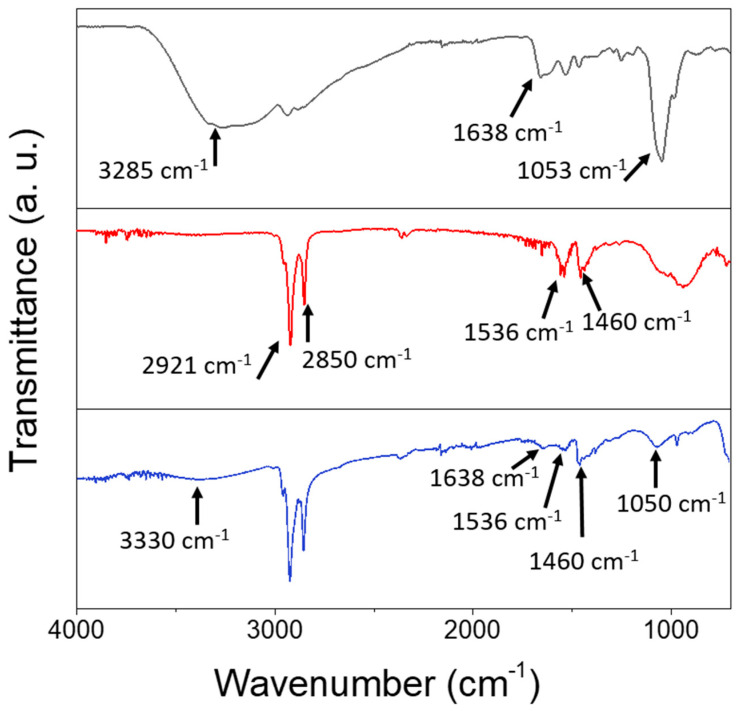
FTIR-ATR spectra of PDA (black line), SPIONs (red line) and SPION@PDA nanoparticles (blue line), cast from aqueous solution.

FTIR spectroscopic characterization was performed on the prepared SPION@PDA NPs and compared to those of the as-prepared SPIONs and the bare PDA (Figure 4). The spectral line shape, though similar for the SPIONs before and after PDA functionalization, exhibits distinctive differences. Indeed, both spectra are characterized by the presence of the peaks at 2927 cm^−1^, ascribable to the asymmetric stretching vibration of (-CH_2_) and of the peak at 2850 cm^−1^ due to the symmetric stretching of -CH_3_ metric. Well-defined peaks at 1536 cm^−1^ and at 1460 cm^−1^, respectively, can be attributed to the symmetric and asymmetric stretching frequency of C=O of the bidentate bond of the carboxylate anion coordinating the SPION surface [44]. Such characteristic bands can be due to the capping agents (OLEA) used for the synthesis procedure and expected to coordinate the surface of the SPIONs. A broad band observed in a region from 3500 to 3000 cm^−1^, both in the PDA and SPION@PDA NP spectra, can be ascribed to the stretching vibration of the hydroxyl and N-H groups of the PDA, while the absorption peak at 1050 cm^−1^ could be due to the C-N stretching vibration in the PDA. Finally, the peak at 1638 cm^−1^, present both in the PDA and SPION@PDA NP spectra, can be assigned to N-H vibrations. The overall data indicate that, while the original coordinating molecules are still present, the PDA functionalization of the prepared NPs has successfully taken place.

**Figure 5:**
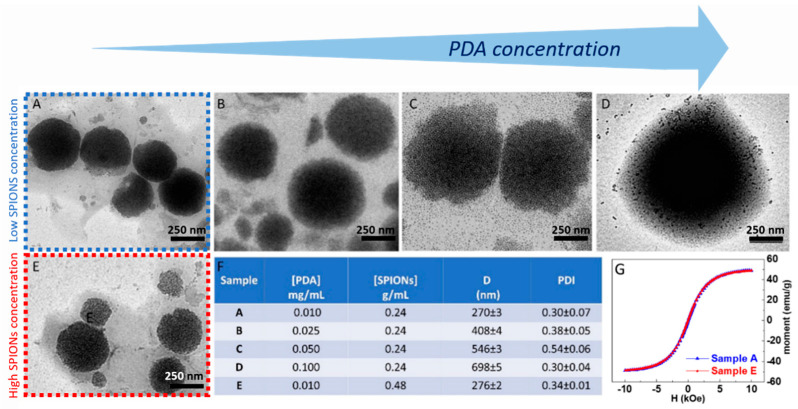
TEM micrographs obtained with staining (staining time of 30 s) of SPION@PDA NPs prepared at SPION concentration of 0.24 g/mL and PDA concentration of 0.01 mg/mL (**A**), 0.025 mg/mL (**B**), 0.05 mg/mL (**C**), 0.1 mg/mL (**D**), respectively, and SPION concentration of 0.48 g/mL and PDA concentration of 0.01 mg/mL (**E**). Table reporting the average intensity hydrodynamic diameters and the corresponding polydispersity index (PDI) for each SPION/PDA prepared sample. All reported data are expressed as mean values ± SD of three replicates (**F**). RT hysteresis cycle of sample A (blue line) and B (red line) (**G**).

The preparation of the superparamagnetic nanostructures was performed by optimizing the synthetic parameters and investigating the effect of the PDA/SPION weight ratio on the final morphology of the nanostructures. The effect in the synthesis of the two parameters, namely the amount of PDA and SPIONs, on the final morphology of the PDA-coated SPIONs was then considered. Firstly, the effect of the concentration of PDA was investigated by varying its concentration in a range from 0.01 to 0.1 mg/mL and keeping the concentration of SPIONs constant at 0.24 g/mL. TEM micrographs obtained by staining the sample (Figure 4) show that the PDA functionalization of SPIONs turns in the formation of well-isolated PDA/SPION NPs, with a multicore structure formed of clustered SPIONs, characterized by spherical morphology and size, depending on the starting PDA concentration. In particular, for a PDA concentration value of 0.01 mg/mL (Figure 5A), the formation of spherical magnetic clusters with an average diameter of (270 ± 3) nm was observed. Increasing the PDA concentration up to 0.025 mg/mL (Figure 5B), nanostructures with an average diameter of (408 ± 4) nm are observed, while a 0.05 mg/mL PDA concentration (Figure 5C) results in an increment in the average diameter up to (546 ± 3) nm. Finally, for the higher tested PDA concentration (0.1 mg/mL), the nanostructures present an average diameter of (698 ± 4) nm and are characterized mostly by spherical shape.

The obtained results allow one to define the best experimental conditions, that is, a PDA concentration of 0.01 mg/mL, suitable for the preparation of quite homogeneous magnetic PDA NPs, with highly spherical morphology and an average diameter lower than 300 nm, ensuring a high surface-to-volume ratio useful for the efficient removal of heavy metal ions from an aqueous environment.

**Figure 6:**
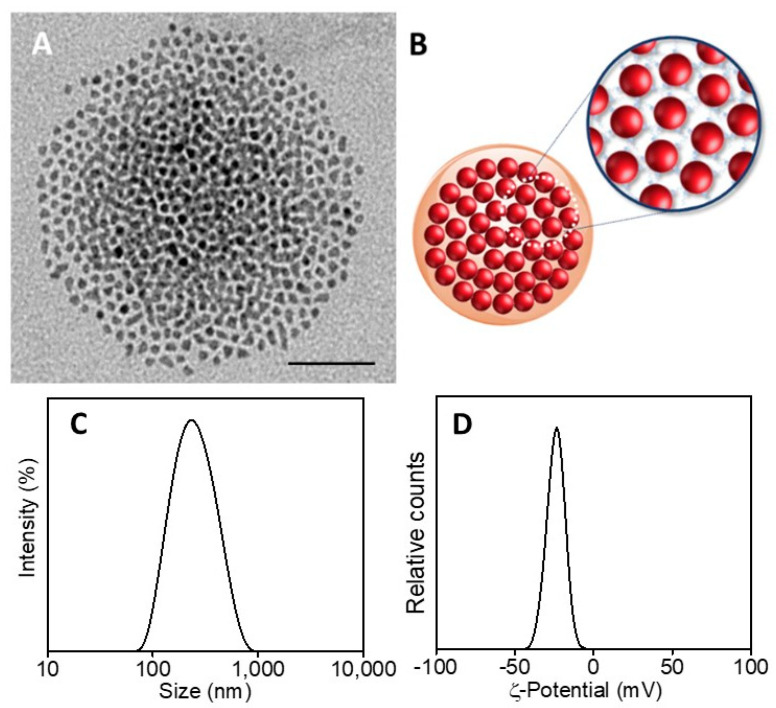
TEM micrograph of SPION@PDA NPs, dispersed in aqueous solution (scale bar 50 nm) (**A**). Representative sketch of SPION@PDA NPs (**B**). Intensity–size distribution by DLS analysis (**C**) and ζ-Potential curve (**D**) of SPION@PDA NPs.

SPION@PDA NPs were also obtained at higher SPION content, increasing the concentration from 0.24 g/mL to 0.48 g/mL and keeping the PDA concentration constant (0.01 mg/mL) to investigate the effect of the SPION cargo on the final magnetic properties of the nanocomposites. After their preparation, ICP AES analysis resulted in an Fe concentration value of about 2 × 10^−4^ M for the two PDA NP samples obtained at the two different starting SPION concentrations. Such a result suggests that the starting SPION concentration of 0.24 g/mL ensures a convenient amount of SPIONs that can be successfully embedded in the SPION@PDA NPs at a PDA concentration of 0.01 mg/mL, while a starting SPION concentration of 0.48 g/mL turns into an excess of SPIONs that cannot be PDA functionalized and that are then removed in the purification steps. Indeed, DLS analysis of the obtained samples confirmed that increasing the SPION concentration from 0.24 g/mL to 0.48 g/mL, no relevant modification in size occurs, with average diameter of the nanoclusters of (270 ±3) nm and (276 ± 2) nm, respectively (Figure 5F). The size measured from TEM micrographs is in a good agreement with that obtained by DLS data (Figure 5). Interestingly, the SPION@PDA NPs, when investigated by TEM without applying any staining procedure (Figure 6), appear to be formed of clustered SPIONs that retain their individuality and maintain a rather constant interparticle distance, thus accounting for the presence of the original coordinating molecules at the SPION surface, which interdigitate their alkyl chains.

The corresponding room-temperature magnetization measurements performed on the two tested SPION/PDA samples revealed a similar magnetic response (Figure 5G), with a magnetic moment reduction with respect to the as-synthesized SPIONs (from ca. 60 emu g^−1^ to 49 emu g^−1^ at 10 kOe) that can be ascribed to the presence of the PDA layer, encapsulating the SPIONs. However, the SPION@PDA clusters still present a superparamagnetic behaviour. These findings are in agreement with magnetization studies on SPION nanoparticles and their nanocomposites [45,46] and confirm that the polymer encapsulation does not affect the magnetic behaviour but causes a reduction in magnetization by reducing the contribution of SPION surface spins to the overall magnetization.

On the basis of the obtained results, the best conditions for the formation of nanostructures, in terms of morphology, size and homogeneity, were found for a PDA concentration of 0.01 mg/mL and a SPION concentration of 0.24 g/mL. Intensity–size distribution by DLS analysis performed on this sample highlights the formation of SPION@PDA NPs characterized by a monomodal and quite homogeneous particle population. The TEM micrograph of the bare, not-stained, samples shows (Figure 5A) a single SPION@PDA NP, clearly indicating that the surface functionalization of SPIONS with PDA induces the formation of spherical PDA NPs embedding multicores of SPIONs (Figure 5C). The ζ-potential value of (−24.1 ± 3.4) mV recorded for SPION@PDA NPs indicates a negative charge of their surface that can be reasonably ascribed to the deprotonation of catechol groups [47] and accounts for their good colloidal stability in aqueous media (Figure 5D).

### 3.2. Adsorption of Cu^2+^ Ions to SPION@PDA Nanoparticles

**Figure 7:**
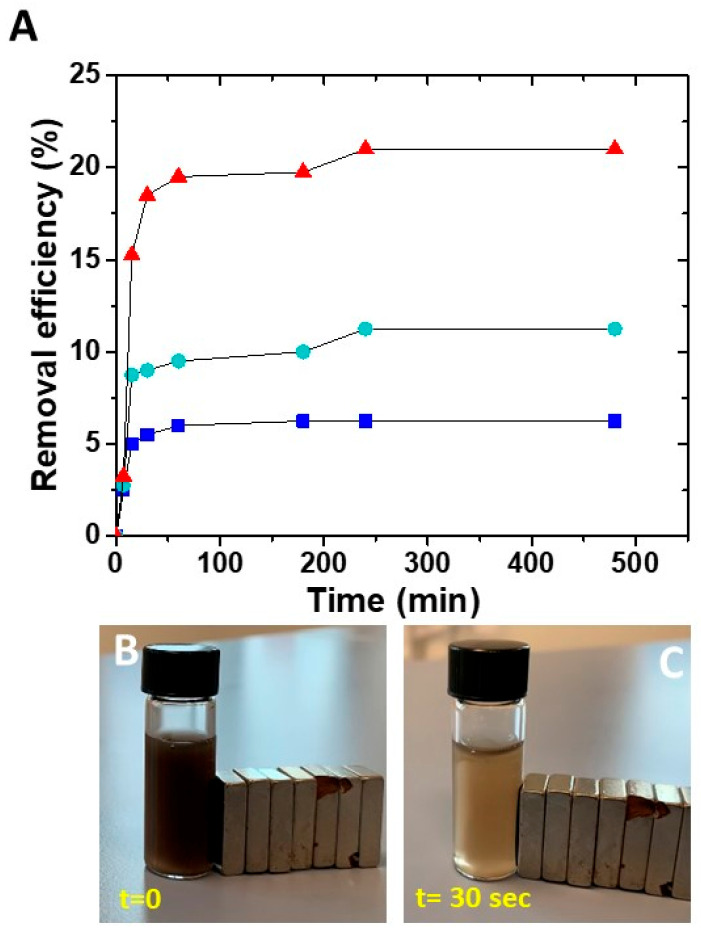
Time course of removal efficiency (%) of Cu^2+^ by SPION@PDA NPs at different concentrations: 0.2 mg/mL (blue line), 0.4 mg/mL (green line), 0.8 mg/mL (red line) (**A**). SPION@PDA NP solution at beginning of the experiment (t = 0) (**B**) and 30 s after (**C**) the application of an external magnetic field.

The effective capability of the prepared nanocomposites in adsorbing the metal ions was evaluated considering different concentrations of SPION@PDA NPs that were dispersed in a Cu aqueous solution containing Cu^2+^ ions at a concentration of 5 ppm for 8 h. The removal efficiency was then calculated as previously described (Figure 7). At a fixed Cu^2+^ ion concentration in the aqueous solution, the removal efficiency was different for the diverse investigated sample concentration, suggesting that the affinity of the NPs towards Cu^2+^ ions depends on the SPION@PDA NP concentration and, in particular, on the amount of PDA on the SPION surface. When the SPION@PDA NP concentration increased from 0.2 mg/mL to 0.4 mg/mL and finally to 0.8 mg/mL, the removal efficiency increased accordingly, reaching values equal to 6.25%, 11.25% and 21%, respectively. These data suggest that the concentration of chemical groups, namely the catechol groups, available on the surface of SPIONs determines the amounts of the adsorbed Cu^2+^ ions.

For all the investigated SPION@PDA NP concentrations, the removal efficiency of Cu^2+^ remains steady for contact time higher than about 4 h, as complete saturation of the active sites on the adsorbent surface occurs.

### 3.3. Adsorption Isotherms

In the adsorption, a surface process leads to the transfer of a compound, sorbate, from the fluid bulk to the solid surface of the sorbent. Adsorption isotherms describe the equilibrium relationship occurring between sorbent and sorbate and represent the ratio between the adsorbed quantity of the investigated substance and its portion remaining in solution at a fixed temperature at equilibrium.

Among the different isotherm equations available in the literature, Langmuir isotherms were selected for this study.

The Langmuir isotherm is represented by Equation (3):

*q_e_* = *K_L_q_max_C_e_*/(1 + *K_L_C_e_*) (3)

where *q_e_* (mg/g) and *C_e_* (g/L) are the amount of adsorbed Cu^2+^ ions per unit weight of adsorbent and the unadsorbed Cu^2+^ ion concentration in solution at equilibrium. *K_L_* (L/mg) is the Langmuir constant and expresses the affinity of the sorbate for binding sites, while *q_max_* (mg/g) represents the maximum adsorption capacity when the surface is fully covered with Cu^2+^ ions. The linearized form is represented by Equation (4):

*C_e_/q_e_* = 1/*K_L_q_max_* + *C_e_*/*q_max_* (4)

**Figure 8:**
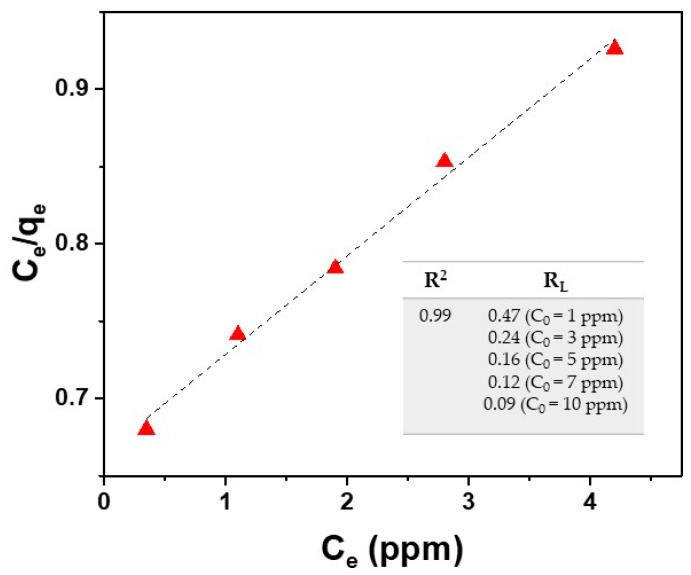
Fitting of experimental data (red triangle) by using the Langmuir isotherm model for Cu^2+^ ions (Equations (3) and (5)).

The plot of *C_e_*/*q_e_* against *C_e_* provides a straight line with slope 1/*q_max_* and intercept 1/*K_L_q_max_.* Langmuir isotherm is based on the assumption that the absorption process has a monolayer nature. As evident in Figure 8, by using the Langmuir isotherm model, the fitting of experimental data deriving from the Cu^2+^ ion adsorption onto the surface of the SPION@PDA NPs, as described in Section 3.1, provides a *q_max_* value of 16.7 mg/g for Cu^2+^ ions (at 298.15 K) and a K_L_ value of 0.096 g/L calculated from the fitting parameters. This model fits the experimental data remarkably well for the tested Cu^2+^ (R^2^ = 0.99).

A dimensionless parameter *R_L_*, called equilibrium parameter and related to the absorption system, was calculated for different concentrations of Cu^2+^ ions. This parameter is given by:

*R_L_* = 1/(1 + *K_L_C*_0_) (5)

As reported in the literature, *R_L_* value indicates the type of isotherm (*R_L_* = 0 irreversible, *R_L_* = 1 linear, *R_L_* > 1 unfavorable, 0 < *R_L_* < 1 favorable) [48]. The results reported in the table of Figure 8 reveal favorable absorption for the studied case.

The obtained results prove that the Langmuir model fits the experimental data, suggesting that the absorption process can be considered as monolayer adsorption with all the reaction sites displaying the same affinity to copper ions.

Sun et al. synthetized Fe_3_O_4_@polydopamine@polyamidoamine (Fe_3_O_4_@PDA@PAMAM) core–shell nanocomposites as adsorbents for Cu(II), finding a q_max_ value of 97.18 mg/g [49]. In another study, Li et al. applied Fe_3_O_4_@Au@polydopamine NPs in the separation and analysis of Cu(II), with a maximum absorption capacity of 37.86 mg/g [50]. Core–shell hollow magnetic NPs (Fe_3_O_4_@PDA) synthetized by Wang et al. [51] exhibited a *q_max_* of 86.35 mg/g, while Culita et al. demonstrated that the adsorption capacity of polyamine-functionalized magnetic NPs decreased as the length of polyamine chain increased, with values ranging from 39.2 to 52.3 mg/g [52]. The here-presented SPION@PDA NPs show a maximum adsorption capacity of 16.7 mg/g, as calculated by fitting our experimental data, which represents a satisfactory value, though lower than those reported for the similar nanoadsorbents (iron oxide/polydopamine). Therefore, in order to apply these NPs as promising and efficient adsorbents for Cu(II) uptake, an optimization of the absorbent properties of the system needs to be further addressed in future work, to improve its removal efficiency.

## 4. Conclusions

In the present work, novel SPION@PDA clusters in the sub-micrometer range were designed, prepared and comprehensively characterized by means of complementary optical, structural and magnetic techniques, to be exploited as adsorbents for Cu^2+^ ions from water. The resulting magnetic NPs, characterized by a very regular morphology, with SPIONs arranged in an ordered assembly and at a regular interparticle distance, featured amino groups at their surface and were tested for copper ion removal from an aqueous matrix.

The uptake efficiency was calculated for different concentrations of SPION@PDA NPs and the Langmuir isotherm study provided a maximum absorption capacity at room temperature of 16.7 mg/g for Cu^2+^ ions, pointing out the potential application of the SPION@PDA NPs as efficient nano-adsorbents for the removal of copper ions from aqueous matrices. The optimization of the adsorption properties of the system needs to be further addressed in future work, extending the study to real water samples and to the effect of other variables, such as the ionic strength, pH and the Cu^2+^ concentration, on the adsorption capacity, thus fully assessing the potential of these functional nanostructures as cost-effective and environmentally friendly solutions.

## Data Availability

The data presented in this study are available on request of the corresponding author.
